# Polyethyleneglycol-Betulinic Acid (PEG-BA) Polymer-Drug Conjugate Induces Apoptosis and Antioxidation in a Biological Model of Pancreatic Cancer

**DOI:** 10.3390/polym15020448

**Published:** 2023-01-14

**Authors:** Karabo Sekopi Mosiane, Ekene Emmanuel Nweke, Mohammed Balogun, Pascaline Nanga Fru

**Affiliations:** 1 Department of Surgery, School of Clinical Medicine, Faculty of Health Sciences, University of the Witwatersrand, 7 York Road, Parktown, Johannesburg 2193, South Africa; 2 Biopolymer Modification and Therapeutics Lab, Materials Science & Manufacturing, Council for Scientific and Industrial Research, Meiring Naude Road, Brummeria, Pretoria 0001, South Africa

**Keywords:** Polyethyleneglycol-betulinic acid, conjugation, apoptosis, antioxidation, reactive oxygen species

## Abstract

Pancreatic cancer (PC) is one of the most aggressive solid malignancies with poor treatment response and low survival rates. Herbal medicines such as betulinic acid (BA) have shown potential in treating various solid tumours, but with limitations that can be circumvented by polymer-drug conjugation. Polyethylene glycol-BA (PEG-BA) polymer-drug conjugate has previously shown selective anticancer activity against PC cells. Here, we elucidate the mechanism of cell death and the cell death pathway, anti-inflammatory and antioxidant activities of PEG-BA. PEG-BA induced apoptotic cell death by arresting MIA-PaCa-2 cells in the Sub-G_1_ phase of the cell cycle compared with BA and untreated cells (39.50 ± 5.32% > 19.63 ± 4.49% > 4.57 ± 0.82%). NFκB/p65 protein expression was moderately increased by PEG-BA (2.70 vs. 3.09 ± 0.42 ng/mL; *p* = 0.1521). However, significant (*p* < 0.05) overexpression of the proapoptotic genes *TNF* (23.72 ± 1.03) and *CASPASE 3* (12,059.98 ± 1.74) compared with untreated cells was notable. The antioxidant potential of PEG-BA was greater (IC_50_ = 15.59 ± 0.64 µM) compared with ascorbic acid (25.58 ± 0.44 µM) and BA-only (>100 µM) and further confirmed with the improved reduction of hydroperoxide levels compared with BA-only (518.80 ± 25.53 µM vs. 542.43 ± 9.70 µM). In conclusion, PEG-BA activated both the intrinsic and extrinsic pathways of apoptosis and improved antioxidant activities in PC cells, suggesting enhanced anticancer activity upon conjugation.

## 1. Introduction

Pancreatic cancer (PC) is a lethal solid malignancy with increasing incidence and mortality worldwide [1]. Most patients present with advanced stages of the disease, in which case the tumour is inoperable and has metastasised to other sites or both [2]. Currently, the best curative option is surgery using adjuvant chemotherapy, with only 15% of patients presenting with resectable or early-stage disease [2]. Combination chemotherapy regimens, including gemcitabine and FOLFIRINOX (oxaliplatin, irinotecan, fluorouracil and leucovorin), are used as the first-line therapy for PC and have shown improved patient survival [3]. Although survival has improved, this is still insufficient as the survival rate is still abysmal compared with other cancers. Furthermore, studies have demonstrated that the administration of chemotherapy could lead to adverse side effects suggesting the need to develop potent drugs against PC but with reduced side effects, including toxicity to normal cells [4].

Betulinic acid (3β-hydroxy-lup-20(29)-en-28-oic acid), the carboxylic acid derivative of betulin, is a naturally occurring, widely distributed pentacyclic triterpene sourced from the outer bark of the Betula alba tree [5]. Betulinic acid (BA) has shown promising anticancer efficacy against several tumours, including cervical cancer, melanomas, leukaemia, blastomas and PC [6,7,8]. However, like any other natural compound, BA is associated with several limitations, including a high molecular weight, which inhibits passage through the lipid bilayer of cells, and poor solubility, which reduces biodistribution, bioavailability and overall therapeutic index [9].

Using nanoparticles in chemotherapy provides an efficient and selective drug delivery system for targeting tumour cells [10]. An example of this type of platform is polymer-drug conjugates, macromolecular constructs covalently linked to a polymeric carrier. Drug conjugation is known to maximise efficacy, reduce adverse side effects, prolong blood circulation, passively deliver drugs to tumours via the enhanced permeability and retention (EPR) effect and overcome tumour resistance [11,12]. In a recent study from our laboratory, conjugation of polyethylene glycol (PEG) to BA showed selective toxicity to PC cells compared with BA alone, resulting in increased apoptotic cell death [8]. However, the specific mechanism of apoptotic cell death and possible evasion strategies employed by the cancer cells were not investigated.

Apoptosis occurs via two different pathways, namely the extrinsic (death receptor) and intrinsic (mitochondrial) pathways [7]. The extrinsic pathway involves the formation of the death-inducing signalling complex (DISC) and subsequent activation of procaspase 8 to caspase 8 [13]. In type 1 cells, the signal from caspase 8 is strong enough to activate procaspase 3 to caspase 3 and leads to apoptosis [14,15]. In type 2 cells, the signal is weak and requires amplification via cleavage of Bid (truncated Bid), which is translocated to the mitochondria [7,15,16]. The intrinsic pathway is caused by cellular stress that disrupts the mitochondrial membrane potential, leading to leakage of cytochrome c (Cyt C) into the cytosol via the Bak/Bax channel [7,17,18]. CytC interacts with deoxyadenosine triphosphate (dATP), apoptotic proteinase-activating factor-1 (Apaf-1) and procaspase 9, forming a complex known as the apoptosome, which leads to the autoactivation of procaspase 9 to caspase 9, which in turn activates procaspase 3 to caspase 3, leading to apoptosis [13].

Cancer cells have developed mechanisms to evade apoptosis, a major hallmark of carcinogenesis [19]. One such mechanism is the dysregulation of pro- and antiapoptotic genes or proteins [20,21]. For example, the B-cell lymphoma 2 (Bcl-2) family, which includes both pro (including puma, Bid, Bad, Bax and Bak) and anti (such as Bcl-2, Bcl-XL and Mcl-1)-apoptotic members, are frequently dysregulated in carcinogenesis [21]. Unlike some tumours, PC is associated with the downregulation of the Bcl-2 protein [22,23,24]. This suggests that evasion of apoptosis is induced by the other antiapoptotic proteins (Bcl-X_L_ and Mcl-1). These proteins have been associated with the nuclear factor kappa-light chain enhancer of activated B cells (NF-κB), a mediator of inflammation, which is overexpressed in tumours [22]. Like other cancers, PC is known to downregulate the expression of Bax and Bak, favouring survival over cell death [22,25]. Furthermore, other studies have shown that cancer cells inhibit the activation of caspase proteins by overexpressing the inactive forms compared with the activated caspases [26,27,28].

In the case of cellular injury, the immune system is activated in an attempt to alleviate any resulting potential damage [29,30]. One of the response mechanisms used by the innate immune system is inflammation [29], with reactive oxygen species (ROS) playing a significant role. ROS are necessary for embryonic development at moderate levels, guiding tissue maintenance and immunity [31]. Consequently, chronic inflammation and subsequent overproduction of ROS can result in the induction of DNA oxidation, affecting the stability of the genome and causing gene mutations, which in turn facilitate cancer initiation [29,31,32,33,34]. Furthermore, inflammation aids in the formation of the tumour-promoting microenvironment and can cause chemoresistance in many cancer types, including PC [29,35]. NF-κB mediates inflammation by regulating pro-inflammatory cytokines such as interleukins, transforming growth factor beta and tumour necrosis factor-alpha [29,30,36]. In addition, NF-κB is continuously transcribed in PC, leading to transcriptional and translational upregulation of genes involved in angiogenesis, apoptosis inhibition, tissue invasion and growth factors, activating survival pathways [36,37,38].

In this study, PEG-BA was assessed for its effect on apoptosis by measuring DNA content, proapoptotic gene expression, and NF-κB protein activity on MIA PaCa-2 treated cells. Its effect on the inflammation induced by ROS was also assessed. The findings demonstrated that PEG-BA induces apoptosis by arresting cells in the sub-G_1_ phase of the cell cycle, activates proapoptotic genes of the extrinsic and intrinsic pathways and inhibits ROS formation. Overall, there was cell growth inhibition, apoptosis induction and ROS activity inhibition in PC cells treated with PEG-BA, suggesting that apoptosis is the primary anticancer mechanism.

## 2. Materials and Methods

### 2.1. Compound Preparation

The compounds were synthesised and characterised as previously described [8,39]. The free compound (BA), conjugate (PEG-BA) and standard (gemcitabine) were dissolved to 20 mg/mL in tissue culture grade dimethylsulfoxide (DMSO) (Sigma-Aldrich, St. Louis, MO, USA) and stored in single-use aliquots at −20 °C until required for experiments.

### 2.2. Cell Culture Models and Maintenance

The biological model for PC used in this study was the MIA PaCa-2 cells obtained from the Japanese Collection of Research Bioresources (JCRB) Cell Bank (JCRB Cell Bank; Ibaraki, Japan). These cells were derived from a 65-year-old man with pancreatic adenocarcinoma located in the tail and body of the pancreas [40]. Vero cells, derived from the kidney of the African green monkey (Cercopithecus aethiops), were used as a model for normal cells to compare with the PC cells in the cytotoxicity and antioxidant activity assays.

Both cell lines were cultured in Dulbecco’s modified Eagle’s medium (DMEM) (Sigma-Aldrich, St. Louis, MO, USA) with slight modifications. The DMEM used for the MIA PaCa-2 cells was made up to a final concentration of 13.4 g/L with sodium bicarbonate (NAHCO_3_—3.7 g/L) and 1% antibiotic antimycotic solution (10,000 units/mL of penicillin, 10,000 µg/mL streptomycin and 25 µg/mL amphotericin B). In the case of the Vero cells, the standard DMEM (13.4 g/L DMEM powder + 3.7 g/L NaHCO_3_) was supplemented with 1.1 mM sodium pyruvate, 25 mM HEPES buffer, 250 µg/mL gentamicin, and 0.5% antibiotic antimycotic solution. Both media were further supplemented with 10% heat-inactivated foetal bovine serum (FBS). The cells were cultured in 75 cm^2^ vented flasks (Thermo Fischer Scientific, Waltham, MA, USA) at 37 °C (95% humidity and 5% CO_2_). The cells were harvested every 2–4 days or when they reached 80–90% confluency using a trypsin-EDTA (Sigma-Aldrich, St. Louis, MO, USA) dissociation reagent. Cell counting was performed using the trypan blue exclusion method [41].

### 2.3. Cell Treatment

For experimental purposes, the cells were allowed to adhere for 24 h and then treated with controls and test compounds diluted with DMEM. DMSO (≥ 0.33%) was used as the vehicle control, 0.4 µM doxorubicin and 0.78–100 µM gemcitabine as control drugs, and 0.78–100 µM BA (free compound) and PEG-BA (polymer-drug conjugate) for 72 h in a humidified incubator (95% humidity, 37 °C and 5% CO_2_).

### 2.4. Microscopic Analysis

MIA PaCa-2 cells exposed to varying concentrations (1, 2, 4 and 8 µM) of BA and PEG-BA were analysed using light microscopy. The morphological changes were photographed after 24, 48 and 72 h using the Olympus IX51 microscope (Olympus—Life Sciences Solutions, Tokyo, Japan) at 10 X magnification.

### 2.5. Cytotoxicity Analysis

Cytotoxicity assays were performed using tetrazolium dyes as previously described [8]. The IC_50_ value, the half-maximal inhibitory concentration representing the concentration at which the compounds will inhibit cell viability by 50% [42], was calculated using GraphPad Prism (v8.0.2.263). This was followed by analysing the ratio between the IC_50_ on Vero and MIA PaCa-2 cells, defined as the selectivity index (SI). 

### 2.6. Cell Cycle Status Determination with Propidium Iodide Dye

The single parameter-based propidium iodide staining technique was used to assess the cell cycle status of the cells, pre-and post-treatment with vehicle control (0.33% DMSO), 4 µM of BA and PEG-BA and 4.5 µM of gemcitabine following 72 h of treatment (37 °C; 95% humidity, 5% CO_2_). The cells were harvested using trypsin EDTA and washed (200× *g*). The cell pellet was resuspended with ice-cold 1X PBS followed by dropwise addition of ice-cold 70% ethanol for fixation at −20 °C for at least 24 h before staining. A total of 500 µL of the staining mixture, prepared by dissolving 2 mg of RNase A in 0.1% Triton-X100 reagent in PBS and containing 400 µL of 500 µg/mL propidium iodide dye, was added to each sample and incubated at room temperature for 30 min. Data acquisition commenced at low flow rates (< 300 events/sec) for improved resolution and adequate DNA quantification on the BD LSRFortessa™ Analyser and FACSDiva software (BD Biosciences, Franklin Lakes, NJ, USA). At least 30,000 events were recorded per sample. The fluorescent signal from the samples was detected using the 610/20 band filter pass filter. The generated Flow Cytometry Standard (FCS) files in FACSDiva were imported and analysed using FlowJo v10.8.1 (FlowJo, LLC, Ashland, KY, USA).

### 2.7. Gene Expression Analysis of Apoptotic Genes

#### 2.7.1. Extraction of Total RNA

Total RNA was extracted using the E.Z.N.A.^®^ Total RNA Kit I (Omega Bio-Tek, Norcross, GA, USA) according to the manufacturer’s guidelines. Briefly, 700 µL of guanidium thiocyanate (GTC)-lysis buffer containing 2% β-mercaptoethanol was added to the pelleted cells previously treated with 4 µM BA and PEG-BA and 4.5 µM gemcitabine for 72 h. This mixture was then transferred to an RNA homogeniser mini-column and centrifuged for 60 s at 13,000× *g*. This was followed by adding 700 µL of freshly prepared 70% ethanol and transferring to a HiBind^®^RNA mini-column. The mixture was centrifuged at 10,000× *g* for 60 s until the RNA in-solution was bound to the column. The bound RNA was washed (30 s at 10,000× *g*) with 500 µL of RNA Wash Buffer I. The filtrate was discarded, and the RNA was rewashed (60 s at 10,000× *g*) using a second buffer (RNA Wash Buffer II containing 100% ethanol). This step was repeated once to ensure proper washing of the RNA before drying the HiBind^®^RNA mini-column to remove any residual ethanol (15,000× *g* for 120 s). The HiBind^®^RNA mini-column was transferred to a 1.5 mL Eppendorf tube, and 60 µL of nuclease-free water was added, followed by centrifugation (15,000× *g* for 120 s) to elute the RNA. The eluted RNA was assessed for purity, and the concentration was determined using the Nanodrop 2000 (Thermo Fischer Scientific, Waltham, MA, USA).

#### 2.7.2. Complementary DNA (cDNA) Synthesis

Total RNA (250 ng/µL) was used to make the cDNA template for each sample. The cDNA synthesis step was performed using the ProtoScript II First Strand cDNA Synthesis Kit (New England BioLabs Inc, Ipswich, MA, USA) following the manufacturer’s protocol. Briefly, 20 µL of the reaction mixture was added to respective Eppendorf tubes: 2 µL of d(T)23 VN (50 µM) primers, 10 µL of the ProtoScript II reaction mix (2 X), 2 µL of the Protoscript II enzyme mix (10 X) and 6 µL of the RNA template diluted with the required amount of nuclease-free water to have a uniform final cDNA concentration for all the samples of 1000 ng/µL. The samples were then incubated in a SimpliAmp Thermal Cycler (Thermo Fischer Scientific, Waltham, MA, USA) under the following conditions: 42 °C for one hour, enzyme inactivation at 80 °C for 5 min, and an infinite hold at 4 °C.

#### 2.7.3. Quantitative Real-Time Polymerase Chain Reaction Analysis

Real-time PCR was performed in a 96-well plate format (on ice) according to the MIQE guidelines [43]. Briefly, 5 µL of the PowerUp™ SYBR™ Green Master Mix (2X), 0.5 µL each of forward and reverse primer (sequences shown in Table 1), 2 µL of the cDNA template, and 2 µL of nuclease-free water were added to each well of a 96-well PCR plate, in duplicate. The universal PCR conditions used entailed a UDG activation at 50 °C for 2 min, Dual-lock DNA polymerase activation at 95 °C for 2 min, PCR cycling (40 cycles) of denaturation (95 °C for 15 s), annealing primer-specific temperature range of 50–60 °C for 15 s and extension (72 °C for 60 s). Two reference genes: β-Actin (ACTB) and mitochondrial ribosomal protein L19 (MRPL19), were used to normalise relative fold expression. Fold changes were determined using the 2^−∆∆CT^ method [44]. 

### 2.8. NF-κB/p65 Transcription Factor Assay

The human nuclear factor kappa-B p65 subunit (NF-κB/p65) enzyme-linked immunosorbent assay (ELISA) kit from Elabscience Biotechnology Inc. (Houston, TX, USA) was used to determine the concentration of NF-kB/p65 present in cellular samples after treatment with complete DMEM, 4 µM of BA and PEG-BA, and 4.5 µM gemcitabine (72 h incubation). Cell supernatants and lysates derived from these samples were used to perform the ELISA according to the manufacturer’s protocol with slight modifications. Briefly, 100 µL of the standards (0–10 ng/mL), lysates (diluted 2X with sample diluent), and supernatants were added to respective wells in the protein-coated 96-well plate, in duplicate, sealed, and incubated at 37 °C for 1.5 h. The samples were discarded, and 100 µL of the biotinylated detection antibody working solution was added and incubated for an hour. A wash step was employed by adding 350 µL of the wash buffer, soaking for at least 60 s, and aspirating three times. Horseradish peroxidase (HRP) conjugate working solution (100 µL) was added to each well, sealed, and incubated for 30 min. The plate was washed five times, and 90 µL of the substrate reagent was added to the wells. The plates were sealed and covered in foil to protect them from light. The plate was incubated for at least 15 min, followed by adding 50 µL of the stop solution. The optical density was measured at 450 nm using a Multiscan Sky microplate reader (Thermo Fischer Scientific, Waltham, MA, USA).

### 2.9. ROS Reduction and Potential Antioxidant Analysis

Although the aetiology of PC is not well-known, inflammation has been described as an important risk factor [2,45]. Two assays were used to assess the potential antioxidative capacities of BA and PEG-BA. This was performed using the 2,2-Diphenyl-1-picrylhydrazyl (DPPH) antioxidant assay for the compound activity and the N,N-Dimethyl-p-phenylenediamine (DEPPD) assay for ROS activity in MIA PaCa-2 cells.

#### 2.9.1. DPPH Assay

BA, PEG-BA, and the ascorbic acid standard were tested in the antioxidant activity assay at concentrations in the range 0.78–100 µM. This assay was modified from the 2014 protocol by Mistry and Shah [46]. Briefly, 180 µL of distilled water was added to the first nine rows of a 96 well-plate and 100 µL to the remaining wells. This was followed by adding 20 µL of BA, PEG-BA and ascorbic acid (dissolved in methanol) in the appropriate wells in a fume hood to prevent evaporation of the methanol. A two times serial dilution was performed before adding 100 µL of freshly prepared methanolic DPPH solution to triplicate wells, except for the compound control wells, in the dark. The plates were covered in foil and shaken on a Titertek microplate shaker (Medical EXPO, Marseilles, France) for 10 min. This was followed by an additional 35 min incubation period in the dark. After the incubation, absorbance readings were obtained at 517 nm using a Multiscan Sky microplate reader (Thermo Fisher Scientific, Waltham, MA, USA). 

#### 2.9.2. DEPPD Assay

The DEPPD assay was used to determine reactive oxygen metabolites (hydroperoxide) produced by reactive oxygen species (ROS) in PC cells. The oxidative status and ROS reduction potential of the MIA PaCa-2 cells pre- and post-treatment (72 h) with 0.33% DMSO and optimised working concentrations: 8 µM for BA and PEG-BA and 9 µM gemcitabine, were determined using the DEPPD assay. The assay was performed according to the protocol by William (2012), with modifications to accommodate using cell supernatants instead of the standard urine or blood samples [47]. Briefly, 140 µL of 0.1 M sodium acetate buffer (pH 4.8) was added to a 96-well plate. This was followed by adding 10 µL of 0.39–50 µM hydrogen peroxide standard and cell supernatants of negative control of (media only), vehicle control, BA and PEG-BA and standard gemcitabine. One hundred microlitres of R_1_/R_2_ solution (100 µg/mL DEPPD (R_1_) and 4.37 µM FeSO4 (R_2_) at a ratio of 1:25) was added to all wells except the blank. The plate was covered with foil for at least 1 min and analysed using a Victor™ X3 model 2030 multilabel plate reader (PerkinElmer, Waltham, MA, USA) at a wavelength of 505 nm with a kinetic loop of 30 readings per well. 

### 2.10. Statistical Analysis

The GraphPad Prism Version 8 software (GraphPad, San Diego, CA, USA) was used to determine the statistical significance of the data. To assess whether the data were normally distributed, normality tests such as the Shapiro–Wilk (W), Anderson–Darling (A2*), D’Agostino–Pearson omnibus (K2), and Kolmogorov–Smirnov (distance) that are available in the GraphPad Prism (version 8) software were used. It was assumed that a *p*-value greater than α (0.05) meant that the null hypothesis (H_0_) was accepted, and the data were classified as following a normal distribution. Furthermore, quantile-quantile (Q-Q) plots were assessed with the assumption that plotting predicted residual vs. actual residual forms a roughly straight line for normally distributed data. For two groups (passed normality tests and Q-Q plots), an unpaired Student t-test was performed. A two-way analysis of variance (ANOVA) was performed for more than two groups, followed by multiple comparisons using the Tukey post hoc test, if the data passed the normality tests and Q-Q plots. For data that only passed the normality tests but not the Q-Q plots, the non-parametric Kruskal–Wallis was performed followed by Dunn’s post hoc test. A *p*-value of less than 0.05 was considered statistically significant.

## 3. Results

### 3.1. PEG-BA Causes Cell Rounding in a Dose and Time-Dependent Manner

**Figure 1:**
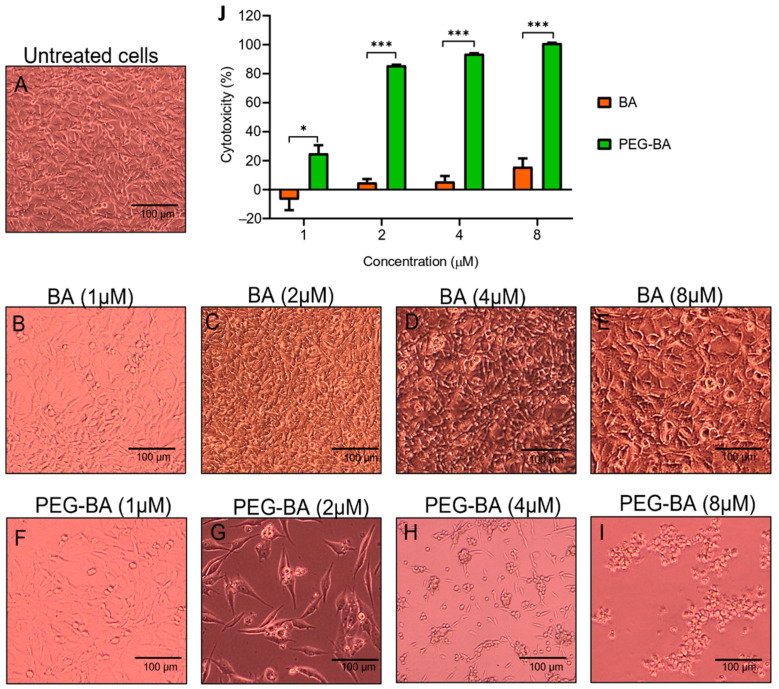
Microscopic and cytotoxic analysis of MIA PaCa-2 cells after treatment with BA and PEG-BA. The cytotoxic effect was measured using MTT tetrazolium dye after 72 h. (**A**) Untreated MIA PaCa-2 cells served as a reference for the morphology of the cells before treatment. (**B**–**E**) MIA PaCa-2 cells were treated with various concentrations (1–8 µM) of BA. The cells continued to grow, even at the highest concentration, although some formed clusters of rounded cells. (**F**–**I**) PEG-BA (1–8 µM) treated MIA PaCa-2 cells. The degree of cells rounding up was dose-dependent, where all the plated cells formed rounded clusters at 8 µM. Magnification: 10 X, scale bar: 100 µm. (**J**) Quantitative bar graph showing cytotoxicity of BA and PEG-BA relative to untreated cells at 72 h (mean ± SEM), n = 3, *: *p* < 0.05 and ***: *p* < 0.001.

**Figure 2:**
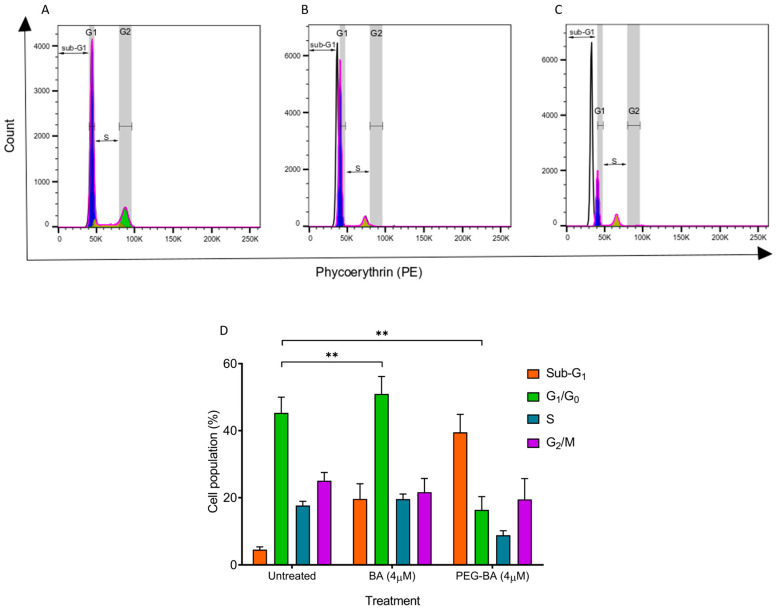
The cell cycle status was monitored using propidium iodide after 72 h of treatment. (**A**–**C**) Representative histograms showing the different phases of the cell cycle indicated by the white (sub-G_1_), purple (G_1_/G_0_), yellow (S) and green (G_2_/M) peaks. Double-sided arrows were inserted to highlight the sub-G_1_ and S phases. Overall, both BA and PEG-BA resulted in a shift of cells from the G_1_/G_0_ to the sub-G_1_ phase, indicating apoptosis induction. (**D**) Quantitative plots representing the overall cycling status of the PC cells (mean ± SEM), n = 3, **: *p* < 0.01. The Inkscape software (Version 1.1) was used to edit Figure 2A–C for clarity.

At least 100,000 cells/mL were visualised for morphological changes before and after treatment using light microscopy. Figure 1A–I show representative micrographs of the effect of BA and PEG-BA on MIA PaCa-2 cells at 72 h only. Quantitative graphs showing percentage cytotoxicity using MTT are shown in Figure 1J. The results showed that, compared with untreated cells (Figure 1A), 2 and 4 µM BA started to induce minimal cell rounding or clumps. However, most plated cells were still viable even at 72 h (4.88 ± 2.42% and 5.55 ± 3.85% cytotoxicity—Figure 1C,D). Cell rounding is associated with apoptosis due to the initial detachment from neighbouring cells and shrinkage [48]. At the highest tested concentration (8 µM), there was a slightly increased cytotoxic effect (15.70 ± 5.70%) that was not visible in the photomicrographs as the cells continued to grow (Figure 1E). On the other hand, the conjugated compound (PEG-BA) resulted in the formation of cell clumps at the lowest tested concentration (1 µM—Figure 1F) and a cytotoxic effect higher than that induced by BA at the same concentration (*p* = 0.0255). Therefore, PEG-BA induced increased cell clumping and cytotoxicity in a concentration and time-dependent manner. At 72 h, 8 µM of PEG-BA resulted in clumping of the cells forming clusters, indicating high toxicity towards the PC cells. This was also observed at 24 and 48 h (Figures S1 and S2). This is further confirmed with a 100% cytotoxicity after 72 h.

### 3.2. PEG-BA Induces a Sub-G_1_ Arrest in Pancreatic Cancer Cells

The cell cycle status of the MIA PaCa-2 cells before and after treatment was performed using propidium iodide dye on 4 µM of BA and PEG-BA treated cells. This is a concentration in the IC_50_ range from this (Figure S3) and a previous study [8]. The untreated MIA PaCa-2 cells showed a high percentage of cells in the G_1_/G_0_ (45.27 ± 4.70%) compared with cells undergoing DNA synthesis (S: 17.67 ± 1.22%) and those preparing to enter mitotic division or already in the phases of mitosis (G_2_/M: 25.07 ± 2.44%) (Figure 2). The remaining cells, which were a minority (4.57 ± 0.82%), were in the area termed the sub-G_1_ phase, which is the phase to the left of the G_1_/G_0_ phase. These results served as baseline populations for comparison after treatment, where a reduction or increase in cells from one phase to another was regarded as due to treatment [49]. At 4 µM, BA shifted the MIA PaCa-2 cells into sub-G_1_ (19.63 ± 4.49%) and induced cell cycle arrest in the G_1_/G_0_ phase relative to the untreated MIA PaCa-2 cells (Figure 2B,D). PEG-BA (4 µM) treatment resulted in a more significant shift of cells into the sub-G_1_ compared with the untreated sample and BA-only (PEG-BA: 39.50 ± 5.32% vs. untreated: 4.57 ± 0.82% and BA: 19.63 ± 4.49%) as shown in Figure 2C,D. Both compounds (PEG-BA and BA-only) inhibited entry into the S and G_2_/M phases. 

### 3.3. PEG-BA Elevates the Expression of Proapoptotic Genes

**Figure 3:**
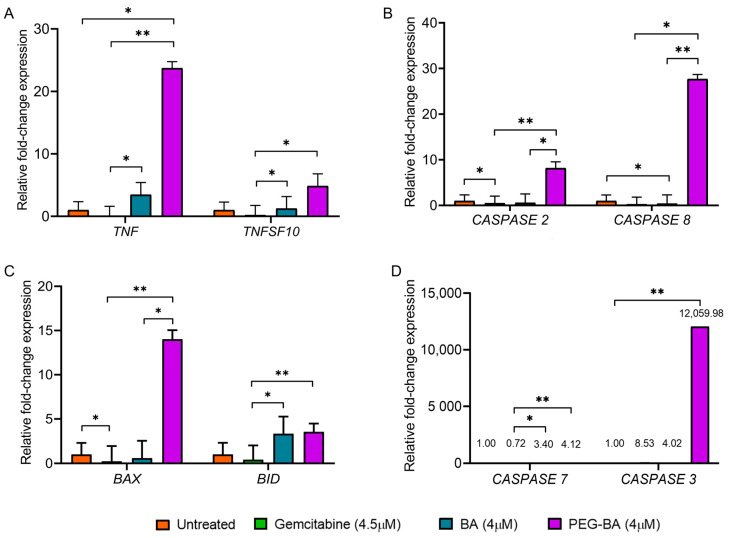
Fold change expression of apoptosis-related genes in treated and untreated MIA PaCa-2 cells. MIA PaCa-2 cells were treated with PEG-BA and BA (4 µM), and the expression of (**A**) *TNF* and *TNFSF10* (**B**), *CASPASE 2* and *CASPASE 8* (**C**) *BAX* and *BID* (**D**) *CASPASE 7* and *CASPASE 3*. Treatment with PEG-BA showed the biggest fold change of *TNF* (23.72 ± 1.03), *TNSF10* (4.87 ± 1.94), *BAX* (14.01 ± 3.55), *CASPASE 3* (12,059.98 ± 1.74), *CASPASE 2* (8.16 ± 1.40) and *CASPASE 8* (27.69 ± 0.99). Expression values are represented as mean ± SD, *: *p* < 0.05 and **: *p* < 0.01.

The cell cycle data reported here and the apoptosis assay performed previously [8] suggest that the anticancer activity of PEG-BA is via apoptosis. We then further investigated the potential mechanism by assessing the expression of key apoptotic genes (*TNF*, *TNFSF10*, *CASPASES* (*2, 3, 7, 8*), *BID* and *BAX*) of the extrinsic and intrinsic pathways of apoptosis using quantitative real-time PCR. Compared with BA, PEG-BA treatment in PC cells resulted in a significant increase in the proapoptotic genes, *CASPASE 2* (0.58 ± 1.93 vs. 8.16 ± 1.40; *p* = 0.0401), *CASPASE 8* (0.40 ± 1.93 vs. 27.69 ± 0.99; *p* = 0.0021) and BAX (0.60 ± 1.93 vs. 14.01 ± 3.55; *p* = 0.0401). Although not significant, *TNF* (3.47 ± 1.95 vs. 23.72 ± 1.03; *p* = 3048), *TNFSF10* (1.24 ± 1.93 vs. 4.87 ± 1.94; *p* > 0.9999), *BID* (3.34 ± 1.93 vs. 3.55 ± 0.93; *p* = 0.9092), *CASPASE 7* (3.40 ± 1.97 vs. 4.12 ± 1.74; *p* = 0.9092) and *CASPASE 3* (4.02 ± 1.93 vs. 12,059.98 ± 1.74; *p* = 0.0872) were also elevated (Figure 3). A reduced expression (*p* > 0.05) of apoptotic genes was observed when the PC cells were treated with gemcitabine, except for the non-significant overexpression of *CASPASE 3* (*p* = 0.0872). Comparing PEG-BA to gemcitabine showed high expression of *TNF* (23.72 ± 1.03 vs. 0.20 ± 1.54; *p* = 0.0021), CASPASE 8 (27.69 ± 0.99 vs. 0.49 ± 1.54; *p* = 0.0401), *BID* (*p* = 0.0087), *CASPASE 2* (8.16 ± 1.40 vs. 0.26 ± 1.55; *p* = 0.0021), *CASPASE 7* (4.12 ± 1.74 vs. 0.72 ± 1.55; *p* = 0.0087), *TNFSF10* (4.87 ± 1.94 vs. 0.06 ± 1.55; *p* = 0.0166) and *BAX* (14.01 ± 3.55 vs. 0.23 ± 1.71; *p* = 0.0021). Furthermore, non-significant overexpression of *CASPASE 3* (12,059.98 ± 1.74 vs. 8.53 ± 1.55; *p* = 0.1712) was also observed.

### 3.4. PEG-BA Treatment May Result in NF-κB Expression in Pancreatic Cancer Cells

**Figure 4:**
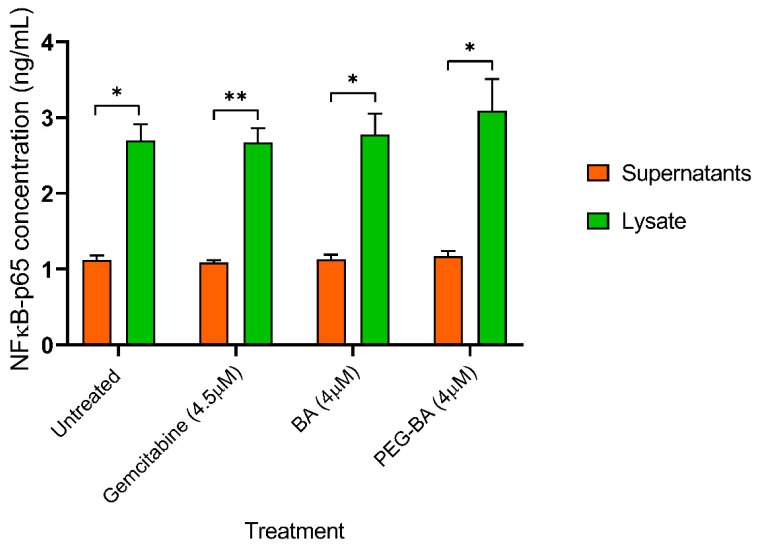
NF-κB protein levels from untreated cell lysates, supernatants, BA and PEG-BA treated MIA PaCa-2 cells. NF-κB levels were higher in the lysate compared with the supernatant. In the lysate, treatment with PEG-BA (3.09 ± 0.42 ng/mL) and BA (2.78 ± 0.27 ng/mL) moderately increased NF-κB levels compared with other groups (untreated: 2.70 ± 0.21 ng/mL and gemcitabine: 2.67 ± 0.19 ng/mL). The date is represented as mean ± SEM, *: *p* < 0.05 and **: *p* < 0.01.

The protein expression levels of nuclear factor kappa B (p65-subunit) in both lysate and supernatant of treated (PEG-BA, BA only and gemcitabine) MIA PaCa-2 cells were quantified using a sandwich ELISA. A higher level of NF-κB was observed in the lysate of the untreated and treated MIA PaCa-2 cells compared with the corresponding supernatant (Figure 4). NF-κB levels remained unchanged in the supernatant (untreated: 1.12 ± 0.06 ng/mL; gemcitabine: 1.09 ± 0.03 ng/mL; BA: 1.13 ± 0.06 ng/mL and PEG-BA: 1.17 ± 0.07 ng/mL), whereas the lysate showed moderate increases with the treatment of PEG-BA (3.09 ± 0.42 ng/mL; *p* = 0.1521) and BA (2.78 ± 0.27 ng/mL; *p* = 0.9981) compared with the untreated sample (2.70 ± 0.21 ng/mL). Gemcitabine resulted in a slight but insignificant decrease (2.67 ± 0.19 ng/mL; *p* = 0.9484). 

### 3.5. PEG-BA Treatment Induces Antioxidant Activities in Pancreatic Cancer Cells

**Figure 5:**
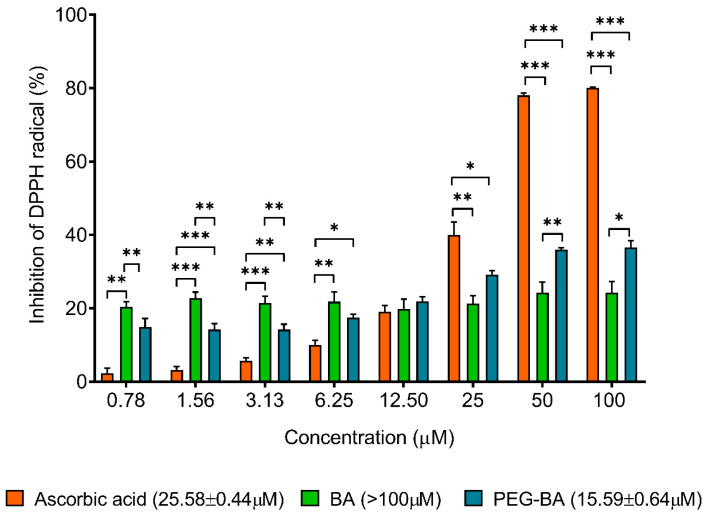
The antioxidant activity of PEG-BA and BA using the DPPH assay. The antioxidant potential was assessed at varying concentrations (0.78–100 µM). The conjugate, PEG-BA, exhibited a dose-dependent antioxidant effect resulting in the highest antioxidant potential compared with ascorbic acid and BA-only. This was indicated by the IC_50_ values shown in brackets on the graph. Data are shown as mean ± SEM (n = 4), *: *p* < 0.05, **: *p* < 0.01 and ***: *p* < 0.001.

Antioxidant analysis using the DPPH assay showed that PEG-BA treatment induced a dose-dependent inhibition of DPPH compared with BA-only (Figure 5). BA treatment did not result in any significant changes in DPPH inhibition for all tested concentrations. At 25 µM, PEG-BA reduced the DPPH radical by 29.17 ± 1.05%, 1.4-fold higher than BA (21.30 ± 2.13%; *p* = 0.1291). This trend was also observed at the remaining concentrations (50 µM: 39.57 ± 0.58 vs. 24.27 ± 2.91% (*p* = 0.0092) and 100 µM: 36.56 ± 1.86% vs. 24.26 ± 3.07%, *p* = 0.0279). In an analysis to determine the compound’s ability to inhibit at least 50% of the initial concentration of the DPPH radical (IC_50_), PEG-BA was found to have the lowest IC_50_ (15.59 ± 0.64 µM) compared with BA-only (>100 µM) and ascorbic acid (25.58 ± 0.44; *p* = 0.006).

**Figure 6:**
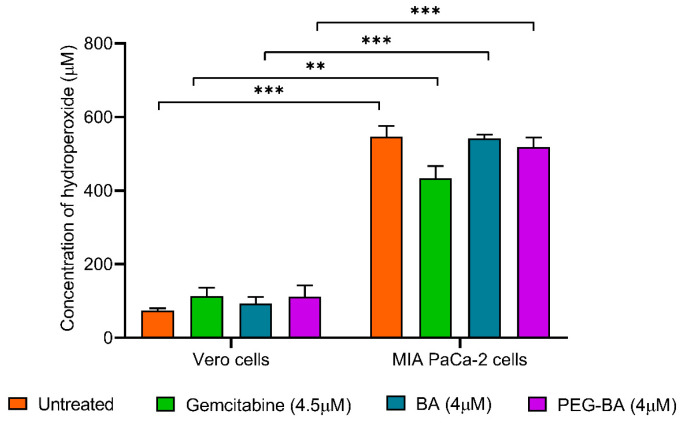
Reactive oxygen species levels were measured as hydroperoxides in MIA PaCa-2 and Vero cells before and after treatment with PEG-BA, BA only and gemcitabine using the DEPPD assay. MIA PaCa-2 cells generally showed higher levels of hydroperoxides compared with the normal cells (546.96 ± 29.03 µM vs. 74.73 ± 5.70 µM, *p* = 0.001). The effect of treatment on the normal cells resulted in a moderate increase in hydroperoxides, especially by gemcitabine (113.65 ± 22.77 µM) and PEG-BA (112.32 ± 29.69 µM) compared with free-BA (93.83 ± 17.43 µM). Conversely, treating the MIA PaCa-2 cells with gemcitabine (433.34 ± 33.53 µM), PEG-BA (518.80 ± 25.53 µM) and free-BA (542.43 ± 9.70 µM) resulted in hydroperoxide reduction compared with the untreated sample. Data are shown as mean ± SEM (n = 3), **: *p* < 0.01 and ***: *p* < 0.001.

The level of ROS, specifically hydroperoxides, in the MIA PaCa-2 cells before and after treatment with PEG-BA, BA only and gemcitabine was assessed using the DEPPD assay. Overall, the results showed that there was a significantly (*p* < 0.0001) higher concentration of hydroperoxides (7.32-fold) in untreated cancer cells compared with the normal cells and treated cells (Figure 6). Treating the MIA PaCa-2 cells with PEG-BA resulted in a moderate reduction in hydroperoxide levels compared with the untreated (Figure 6), with no statistical significance (*p* = 0.5066). Notably, gemcitabine treatment reduced hydroperoxide levels (433.34 ± 33.53 µM) compared with untreated (546.96 ± 29.03 µM; *p* = 0.0451), PEG-BA treated (518.80 ± 25.53 µM; *p* = 0.2011) and BA-only (542.43 ± 9.70 µM; *p* = 0.0581) treated cancer cells.

## 4. Discussion

Pancreatic cancer is associated with chemoresistance. The conjugation of PEG to BA has been shown to kill PC cells effectively and selectively and could circumvent chemoresistance [8]. Analysing critical cellular processes such as apoptosis, inflammation and antioxidation during treatment with PEG-BA can help delineate the molecular mechanisms associated with treatment with the conjugated drug. In this study, PC cells were treated with PEG-BA and its proapoptotic and antioxidative characteristics were demonstrated compared with the free compound, BA and relevant controls.

MIA PaCa-2 cells were assessed using light microscopy to determine the effect of treatment on morphology. In this study, the characteristic spindle-shaped (elongated) morphology of MIA PaCa-2 cells was observed in the untreated sample [50]. Similarly, most BA-treated cells retained that morphology and the cells continued to grow and were still attached to neighbouring cells. A second population of smaller, rounded-up cells was also observed. It is well-documented that the rounding up and shrinkage of cells are critical characteristic features of initiation and progression into apoptosis [51,52]. This is also associated with an intact plasma membrane with reduced cytoplasm, further indicating an earlier phase of apoptosis and not necrosis [53]. Coricovac and colleagues (2021) tested the effect on the morphology of A375 human melanoma cells using BA (5–50 µM). Their results showed that apoptotic cells (smaller and rounded up) were present in a high percentage at 10 and 50 µM, suggesting that a high concentration of BA is required to visualise sufficient morphological differences to the untreated sample [54]. A similar finding was reported by Xu and colleagues (2014), where at least 30 µM of BA was required to inhibit 50% of Hela cells [55]. In contrast, PEG-BA resulted in a dose-dependent induction of cell rounding and shrinkage, which was confirmed by the cytotoxicity results even at 2 µM, suggesting that conjugation improved not only cytotoxicity but also apoptosis induction (confirmed in the downstream analysis performed in this study).

Cell cycle analysis studies, represented by the DNA content, showed an arrest of the cancer cells in the sub-G_1_ phase after treatment with PEG-BA, relative to the untreated and BA-treated cells, suggesting an increase in apoptosis. Studies have shown that the presence of cells in the sub-G_1_ phase indicates apoptotic cells or the induction of apoptosis [56]. This is because this phase represents hypodiploid cells with a DNA content less than that in the G_1_ phase [56,57]. DNA degradation, a notable characteristic of apoptosis, can be one of the causes of the reduced DNA content, indicating that the cells found in the peak before the G_1_/G_0_ are undergoing apoptosis [56,57]. BA has been shown to induce apoptosis in various tumours, including leukaemia [58], neuroblastoma [59,60], cervical cancer [61], colon cancer [62], multiple myeloma [63] and PC [8]. In this study, both PEG-BA and BA induced a reduction of cancer cells in the S-phase, suggesting inhibition of DNA synthesis. The compounds also reduced the population of cells in the G_2_/M phase, indicative of diminished cell proliferation and viability. Similar findings have been reported using PANC-1 and IGROV-1 cells treated with BA, resulting in either a sub-G_1_ or a G_1_/G_0_ phase arrest [64,65]. Taken together, the findings from this study suggest that the drug conjugate PEG-BA induced increased apoptosis compared with free BA, and inhibited DNA synthesis, reduced cell proliferation and viability of PC cells.

Similar to the cell cycle findings, gene expression analysis further suggested a possible apoptosis-inducing ability of PEG-BA. PEG-BA induced the overexpression of proapoptotic genes of the extrinsic and intrinsic pathways, compared with free BA. According to the Central Dogma of molecular biology, DNA serves as the genetic material that is transcribed into messenger RNA and translated into proteins which are essential for cellular functioning [66]. Therefore, a link exists between mRNA and protein levels. A study by Vogel and Marcotte (2013) showed that there is a 40% correlation between gene expression (mRNA quantification) and protein levels [67]. Hence, in this study, the real-time PCR results were compared with literature findings based on proteins that function in apoptosis to try and predict the potential apoptotic mechanism of PEG-BA in PC cells. In cancer, the expression of antiapoptotic genes is elevated, and proapoptotic genes are downregulated [20,21]. *TNF* and *TNFSF10* were upregulated post-PEG-BA treatment in PC cells (Figure 3A). These genes code for pro-inflammatory cytokines, which act as ligands that bind to death receptors, initiating the extrinsic pathway of apoptosis [15]. The products of *TNF* and *TNFSF10* can be inhibited by binding to decoy receptors such as cFLIP, which are overexpressed in several cancer types and aid cell proliferation, growth and survival [26,27]. The inhibition occurs because of the absence of a death effector domain (DED), used by tumour necrosis factor receptor type 1-associated DEATH domain (TRADD) adaptor molecules and procaspase 8 to form the DISC, whose inhibition disrupts the extrinsic pathway of apoptosis [26,27]. *CASPASE 2* and *8,* which code for initiator caspases 2 and 8, and *CASPASE 3* and *7*, which code for executioner caspases 3 and 7, were also overexpressed after PEG-BA treatment (Figure 3B,D). The initiator caspases are activated by forming complexes that facilitate their hydrolysis, which is necessary for caspase function [13]. The activated initiators then activate the executioner caspases, resulting in apoptosis activation. Both types of caspases are inhibited by the inhibitors of apoptosis (IAPs), which are generally overexpressed in cancer cells to favour survival and evade cell death [68,69]. The function of Bid protein in type 2 cells with low caspase signal is necessary for the activation of the mitochondrial pathway, either by direct inhibitory binding to the Bcl-2 protein, allowing Bax/Bak oligomerisation or directly triggering the release of Cyt C [70,71,72]. In this study, PEG-BA resulted in the overexpression of *BAX* and *BID* in the MIA PaCa-2 cells. In most cancers, including PC, both proapoptotic proteins are inhibited by high expression of Bcl-2 antiapoptotic proteins, facilitating the evasion of apoptosis [73]. A study by Naseri and colleagues (2015) showed apoptosis could be restored in cancer cells such as HepG2, T47D and HCT116 by activating Bax [74]. Conjugating PEG to BA improved the apoptosis-inducing ability of the free drug by activating proapoptotic genes. 

It is well-documented that NF-κB protein subunits play an important role in inflammation, cell differentiation, proliferation, and death [75,76,77]. At rest, NF-κB is inactivated by the bound inhibitor IKB protein family preventing its translocation to the nucleus [76]. Phosphorylation inhibits IKBs and releases NF-κB, leading to dimerisation of its subunits (canonical: p50 and p65 or non-canonical: p100 and p52), which is translocated to the nucleus and activates transcription of their target genes [76,78]. In this study, NF-κB levels in the cell lysate were higher than in the supernatant, confirming its intracellular location and that disruption of the plasma membrane was necessary to release adequate amounts of the protein [76]. A slight but insignificant increase in NF-κB was observed after treatment with PEG-BA compared with untreated cancer cells. BA has been shown to activate NF-κB by inducing phosphorylation of the IKBs [6,65]. Notably, the present study showed an upregulation of TNF, an activator of NF-κB [74,75], with PEG-BA treatment. Although some studies have demonstrated the role of NF-κB in inducing cell survival pathways in several malignancies [73], several others have determined its role in apoptosis, specifically its p65 subunit [74,75,76,77]. NF-κB is necessary for BA-induced apoptotic cell death [65]. Ryan et al. (2000) demonstrated the proapoptotic role of NF-κB in Soas-2 cells. They found that p53 (a protein also increased in MIA PaCa-2 cells) induces translocation of NF-κB to the nucleus, thereby activating binding to DNA [79]. In addition, inhibiting NF-κB reduced apoptosis, suggesting that NF-κB may be crucial in the induction of apoptosis by doxorubicin in Soas-2 cells [79]. Another study showed that the activation of the NF-κB/p65 by aspirin resulted in the induction of Bax-dependent apoptosis in human colorectal carcinoma cells [80]. It is worth noting that in this study, PEG-BA treatment resulted in overexpression of *BAX* in the PC cells. These findings may suggest that NF-κB plays an important proapoptotic role, which may be improved by PEG-BA treatment.

PEG-BA treatment further improved antioxidant potential, demonstrated by a lower IC_50_ (15.59 ± 0.64 µM), suggesting a higher radical scavenging potential than ascorbic acid and BA. On the other hand, treatment with BA resulted in an IC_50_ greater than 100 µM, suggesting a lower antioxidant potential than ascorbic acid with an IC_50_ of 25.58 ± 0.44 µM. A similar observation was made by Adesanwo and colleagues (2013), where they assessed the antioxidant capacity of BA isolated from Tetracera potatoria roots and reported an even higher IC_50_ value of 309 µM [81]. Several studies have reported that medicinal plants possess antioxidant activities with desirable radical scavenging abilities [81,82]. This is due to the presence of phenolic groups and tocopherols that can readily donate hydrogen atoms, which is an essential aspect of the DPPH assay, as it is based on the electron transfer ability of potential antioxidants [81,83]. Therefore, with an increase in the number of hydroxyl groups (-OH) or other hydrogen donating groups (-NH, -SH, etc.) in the molecular structure of a compound, the higher the antioxidant activity [81]. Our results, therefore, demonstrate that conjugating PEG to BA improves antioxidant activities due to the inherent anti-inflammatory property of BA, but also with added advantages such as improved solubility associated with conjugation.

The DEPPD assay confirmed significantly high concentrations of hydroperoxides in the PC cells compared with the normal cells (Figure 6), which has also been shown in other studies [32]. A moderate decrease in hydroperoxide concentrations was observed in PEG-BA-treated cancer cells compared with untreated cells. The reduction may be indicative of antioxidant activities [84]. Anticancer compounds possessing antioxidant potential are ideal due to the added advantage of combatting inflammation (ROS), potentially blocking cancer progression [31,32,33]. Our group has previously shown that PEG-BA downregulates the expression of Th1/Th2/Th17 cytokines involved in inflammatory processes, further confirming the anti-inflammatory ability of the conjugate [8,9].

## 5. Conclusions

This study has demonstrated that the potential therapeutic efficacy of betulinic acid on PC cells improved following polymer conjugation. It also showed at the morphological and molecular level that this increased efficacy is due to its ability to upregulate proapoptotic genes and, consequently, induce apoptosis while exhibiting antioxidation properties. These findings suggest that PEG-BA is further investigated as a treatment for PC using models such as 3D culture or animal models.

## Figures and Tables

**Table 1:** List of genes used for real-time polymerase chain reaction.

Gene Name	Forward Primer Sequence	Reverse Primer Sequence
*TNF*	5′-TGCACTTTGGAGTGATCGGC-3′	5′-TTGTCACTCGGGGTTCGAGA-3′
*TNFSF10*	5′-TTGGGACCCCAATGACGAAGA-3′	5′-TGGTCCCAGTTATGTGAGCTG-3′
*BAX*	5′-CCAGCAAACTGGTGCTCAAGG-3′	5′-ACAGGGACATCAGTCGCTTCA-3′
*BID*	5′-GACCCTGGGAGAGCTCTGAAGC-3′	5′-CTCCGACTCACTCCTGGTTCAC-3′
*CASPASE 2*	5′-TACTCCCACCGTTGAGCTGT-3′	5′-TGCCAGCTGGAAGTGTGTTTG-3′
*CASPASE 3*	5′-TTGGAACCAAAGATCATACATGGAA-3′	5′-TGAGGTTTGCTGCATCGACA-3′
*CASPASE 7*	5′-AGTGGATGCTAAGCCAGACCG-3′	5′-TCGAACGCCCATACCTGTCA-3′
*CASPASE 8*	5′-ATAGGCCTGTGACGAAGGTGC-3′	5′-GCGGAATGTAGTCCAGGCTCA-3′
*ACTB* ^1^	5′-CTTCGCGGGCGACGAT-3′	5′-CCACATAGGAATCCTTCTGACC-3′
*MRPL19* ^1^	5′-CCTAGGCCGGAGTTTCCAA-3′	5′-GACTCAAGAACCTGCGTTCC-3′

^1^ The housekeeping genes used to normalise fold expression.

## Data Availability

The data presented in this study are available on request from the corresponding author.

## Supplementary Materials

The following supporting information can be downloaded at: https://www.mdpi.com/article/10.3390/polym15020448/s1, Figure S1: Microscopic analysis of BA and PEG-BA treatment on MIA PaCa-2 cells at 24 h. Figure S2: Microscopic analysis of BA and PEG-BA treatment on MIA PaCa-2 cells at 48 h. Figure S3: Cytotoxic effect of BA and PEG-BA on Vero and MIA PaCa-2 cells.
