# Supplementary material for: Polyethyleneglycol-Betulinic Acid (PEG-BA) Polymer-Drug Conjugate Induces Apoptosis and Antioxidation in a Biological Model of Pancreatic Cancer
Source: Polymers (Basel). 2023 Jan 14;15(2):448. doi: 10.3390/polym15020448 (PMC9863557; doi:10.3390/polym15020448)
Supplement: Supplementary file 1 [file polymers-15-00448-s001.zip › polymers-2024267-supplementary.pdf]

Supplementary Materials

Polyethyleneglycol-Betulinic Acid (PEG-BA) Polymer-Drug Conjugate Induces Apoptosis and Antioxidation in a Biological Model of Pancreatic Cancer

Karabo Sekopi Mosiane [1], Ekene Emmanuel Nweke [1], Mohammed Balogun [2] and Pascaline Nanga Fru [1,*]

[1] Department of Surgery, School of Clinical Medicine, Faculty of Health Sciences, University of the Witwatersrand, 7 York Road, Parktown, Johannesburg 2193, South Africa
[2] Biopolymer Modification and Therapeutics Lab, Materials Science & Manufacturing, Council for Scientific and Industrial Research, Meiring Naude Road, Brummeria, Pretoria 0001, South Africa
* Correspondence: pascaline.fru@wits.ac.za; Tel.: +27-11-717-2476, Fax: +27-11-484-2117

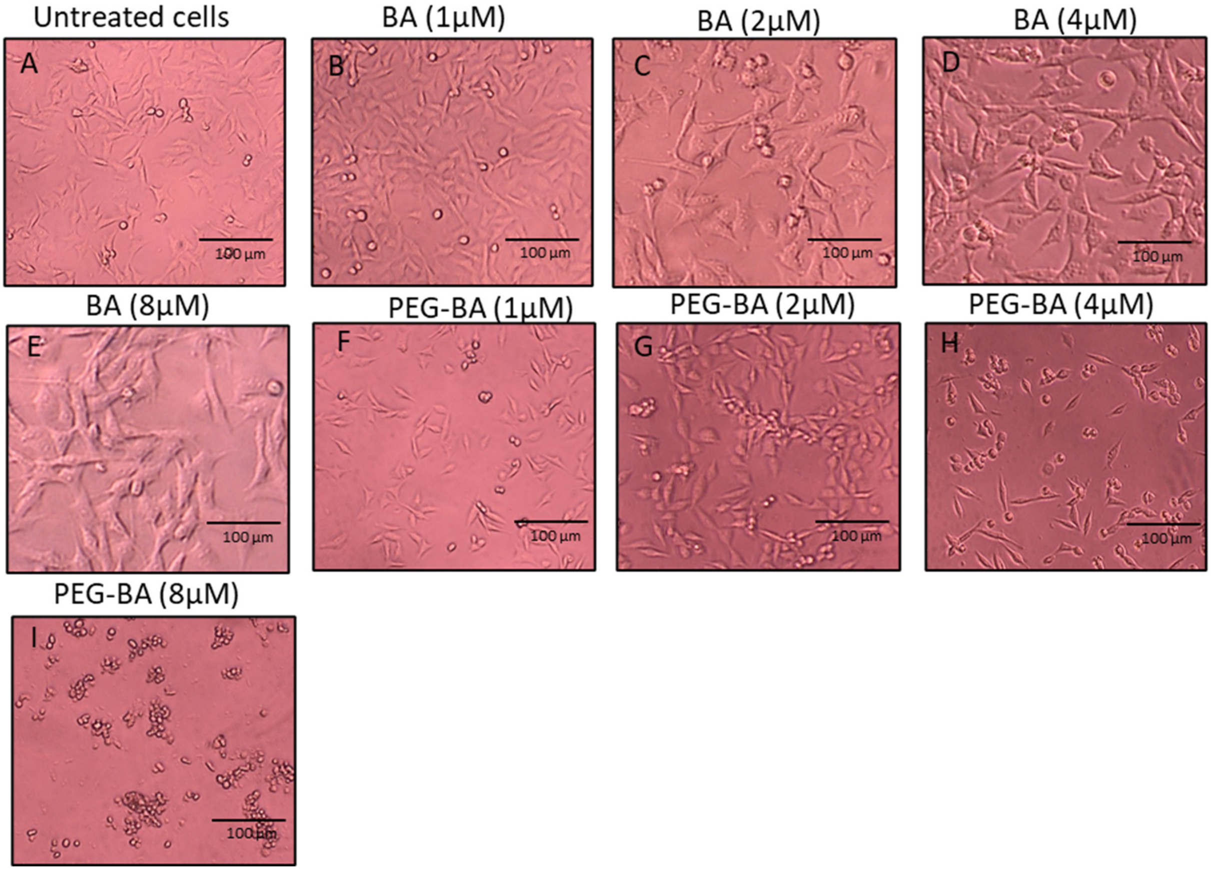

Figure S1. Microscopic analysis of BA and PEG-BA treatment on MIA PaCa-2 cells at 24 h. Untreated cells (A) and cells that were treated with varying concentration (1-8 μM) of native BA (B-E) and its conjugate (PEG-BA) (F-I) were analyzed for morphological changes using light microscopy. Compared to the untreated and BA-treated cells, PEG-BA treated cells had a higher percentage of cells that formed clusters of rounded cells, in a dose-dependent manner.

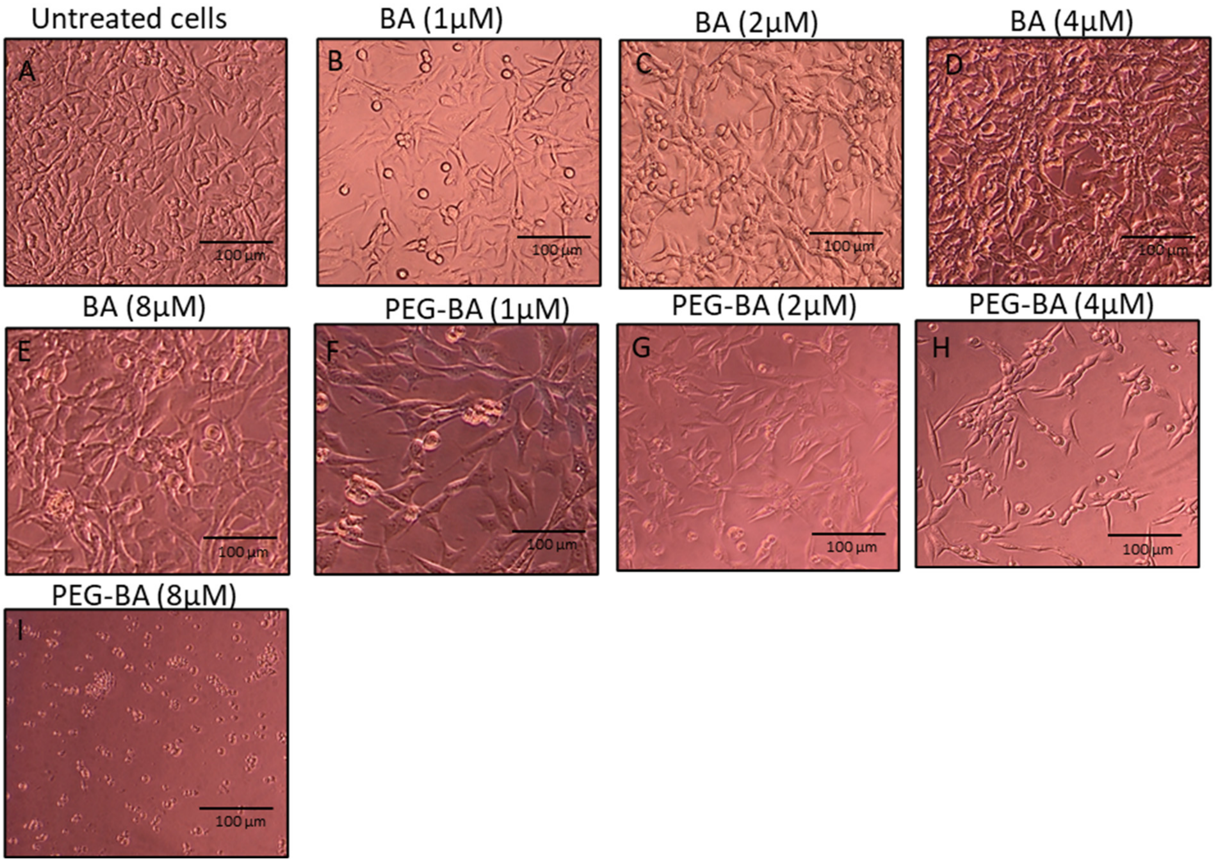

Figure S2. Microscopic analysis of BA and PEG-BA treatment on MIA PaCa-2 cells at 48 h. Untreated cells (A) and cells that were treated with varying concentration (1-8 μM) of native BA (B-E) and its conjugate (PEG-BA) (F-I) were analyzed for morphological changes using light microscopy. Like 24 h, at 48 h, PEG-BA resulted in most of the plated cells forming rounded up cells compared to BA-only, in a dose-dependent manner. This also indicated that PEG-BA induces a toxic effect onto the MIA PaCa-2 cells in a time-dependent manner, since at 48 h, most of the cells start rounding up even at 4 μM.

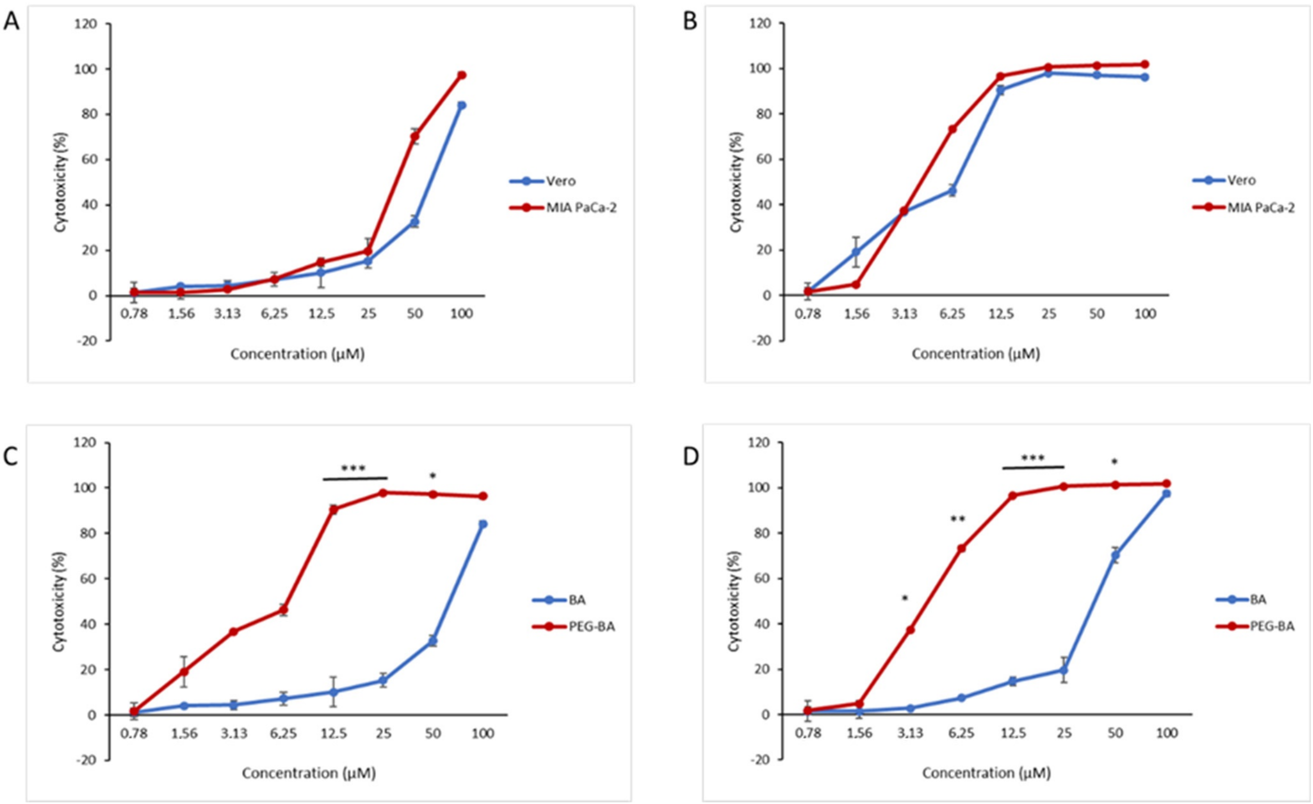

Figure S3. Cytotoxic effect of BA and PEG-BA on Vero and MIA PaCa-2 cells. Cells that were treated with varying concentration (0.78-100 μM) of native BA (A) and its conjugate (PEG-BA) (B) were analysed for cytotoxicity using (XTT) assay. PEG-BA resulted in a higher induction of cytotoxicity than free BA from 12.5-50 μM for Vero cells (C) and from 3.13-50 μM for MIA PaCa-2 cells (D). On both cell lines, BA required at least 25 μM to induce cytotoxicity ≥ 20% (A). With lower IC_{50} values for MIA PaCa-2 cells compared to Vero cells (PEG-BA: 3.01±0.62 μM vs 9.02±0.79 μM and BA: 40.29±3.60 μM vs 45.06±6.27 μM). Data represented as mean±SEM (n=4), *: $p < 0.05$, **: $p < 0.01$ and ***: $p < 0.001$).
